# Effect of high-flow oxygen versus T-piece ventilation strategies during spontaneous breathing trials on weaning failure among patients receiving mechanical ventilation: a randomized controlled trial

**DOI:** 10.1186/s13054-022-04281-w

**Published:** 2022-12-23

**Authors:** Hong Yeul Lee, Jinwoo Lee, Sang-Min Lee

**Affiliations:** 1 Department of Critical Care Medicine, Seoul National University Hospital, Seoul, Republic of Korea; 2 Division of Pulmonary and Critical Care Medicine, Department of Internal Medicine, Seoul National University Hospital, Seoul National University College of Medicine, 101 Daehak-Ro, Jongno-Gu, Seoul, 03080 Republic of Korea

**Keywords:** Extubation, High-flow oxygen, Reintubation, Spontaneous breathing trial, T-piece, Weaning

## Abstract

**Background:**

A spontaneous breathing trial (SBT) is used to determine whether patients are ready for extubation, but the best method for choosing the SBT strategy remains controversial. We investigated the effect of high-flow oxygen versus T-piece ventilation strategies during SBT on rates of weaning failure among patients receiving mechanical ventilation.

**Methods:**

This randomized clinical trial was conducted from June 2019 through January 2022 among patients receiving mechanical ventilation for ≥ 12 h who fulfilled the weaning readiness criteria at a single-center medical intensive care unit. Patients were randomized to undergo either T-piece SBT or high-flow oxygen SBT. The primary outcome was weaning failure on day 2, and the secondary outcomes were weaning failure on day 7, ICU and hospital length of stay, and ICU and in-hospital morality.

**Results:**

Of 108 patients (mean age, 67.0 ± 11.1 years; 64.8% men), 54 received T-piece SBT and 54 received high-flow oxygen SBT. Weaning failure on day 2 occurred in 5 patients (9.3%) in the T-piece group and 3 patients (5.6%) in the high-flow group (difference, 3.7% [95% CI, − 6.1–13.6]; *p* = 0.713). Weaning failure on day 7 occurred in 13 patients (24.1%) in the T-piece group and 7 patients (13.0%) in the high-flow group (difference, 11.1% [95% CI, − 3.4–25.6]; *p* = 0.215). A post hoc subgroup analysis showed that high-flow oxygen SBT was significantly associated with a lower rate of weaning failure on day 7 (OR, 0.17 [95% CI, 0.04–0.78]) among those patients intubated because of respiratory failure (*p* for interaction = 0.020). The ICU and hospital length of stay and mortality rates did not differ significantly between the two groups. During the study, no serious adverse events were recorded.

**Conclusions:**

Among patients receiving mechanical ventilation, high-flow oxygen SBT did not significantly reduce the risk of weaning failure compared with T-piece SBT. However, the study may have been underpowered to detect a clinically important treatment effect for the comparison of high-flow oxygen SBT versus T-piece SBT, and a higher percentage of patients with simple weaning and a lower weaning failure rate than expected should be considered when interpreting the findings.

**Clinical trial registration** This trial was registered with ClinicalTrials.gov (number NCT03929328) on April 26, 2019.

**Supplementary Information:**

The online version contains supplementary material available at 10.1186/s13054-022-04281-w.

## Introduction

A spontaneous breathing trial (SBT) is used widely to determine whether a patient is ready for extubation and liberation from mechanical ventilation [1]. Because both premature and delayed extubation are associated with increased morbidity and mortality, timely liberation from mechanical ventilation is crucial in critically ill patients [2, 3]. The most common strategies used during SBT are T-piece ventilation and low-pressure support ventilation (PSV) [2]. One systematic review of studies that compared different methods for SBT found that T-piece ventilation seems to reflect more accurately the physiological conditions after extubation, whereas PSV reduces respiratory effort compared with T-piece ventilation [4]. A recent randomized controlled trial (RCT) found that the use of a shorter, less demanding SBT strategy led to significantly higher rates of successful extubation [5]. However, although the latest guideline suggests the initial SBT be conducted with low PSV based on limited data [6], choosing the best ventilation strategies during SBT remains a challenging task [4, 7, 8].

High-flow oxygen therapy offers several physiological advantages and may lead to improved comfort and clinical outcomes in patients with hypoxemic respiratory failure [9, 10]. High-flow oxygen therapy is used increasingly for several indications in critically ill patients, including de novo hypoxemic respiratory failure, postextubation, postoperative respiratory failure, and palliative care [9]. Based on the results of RCTs, use of prophylactic high-flow nasal cannula (HFNC) and/or noninvasive ventilation (NIV) after extubation is now used widely and is considered as the first-line prophylactic respiratory support option in patients at risk for reintubation [11–14]. However, there is limited evidence on the efficacy and safety of the high-flow oxygen ventilation strategy during SBT. Moreover, the physiological effects of high-flow oxygen therapy via tracheostomy (HFOT_TRACHEAL_) are lower than HFNC, likely because the tracheostomy connection interface is completely open system [15]. To date, only one recent pilot RCT of patients at high risk of weaning failure showed that the high-flow oxygen SBT neither increased the reintubation rate nor accelerated mechanical ventilation weaning compared with T-piece SBT [16].

In this study, we hypothesized that the high-flow oxygen ventilation strategy during SBT might reduce the rate of weaning failure compared with T-piece ventilation during SBT. Here, we investigated the effect of high-flow oxygen versus T-piece ventilation strategies during SBT on rates of weaning failure among patients receiving mechanical ventilation.

## Methods

### Trial design

From June 2019 through January 2022, a single-center, open-label, parallel-group RCT was conducted in a medical intensive care unit (ICU) at Seoul National University Hospital, which is a 1778-bed tertiary care referral hospital in South Korea. The Institutional Review Board of Seoul National University Hospital approved the study and protocol (approval number H-1904-150-1029). This trial was registered with ClinicalTrials.gov (number NCT03929328). All patients or their legally authorized representatives provided written informed consent to participate in the study.

### Participants

All consecutive adult patients admitted to the medical ICU who required endotracheal intubation and mechanical ventilation underwent screening before enrollment. Patients who were at least 18 years of age were eligible for inclusion if they were receiving mechanical ventilation for at least 12 h, had recovered from the precipitating illness, and had fulfilled the weaning readiness criteria according to international guidelines (Additional file 1: Appendix S1) [1, 17]. The exclusion criteria were tracheostomy and the decision to stop life-supportive therapies.

### Randomization

Patients who were enrolled by the ICU attending physician were randomized in a 1:1 ratio to undergo either the T-piece ventilation strategy or high-flow oxygen ventilation strategy during SBT. T-piece SBT was selected as control group, because it is most commonly used in clinical practice regardless of current guideline and this clinical trial was designed to demonstrate the superiority of high-flow oxygen SBT over T-piece SBT in reducing weaning failure among strategies without using a mechanical ventilator support during SBT [6, 18]. We used a permuted block randomization scheme with computer-generated randomly selected block sizes of 2–6. An independent research nurse maintained the randomization list using sequentially numbered, opaque, sealed envelopes that were inaccessible to clinical investigators. Because the method of ventilation strategy during SBT could not be masked, blinding of the clinical investigators and participants was impossible.

### Interventions

In patients assigned to receive the T-piece ventilation strategy during SBT (T-piece group), the ventilator was disconnected from the endotracheal tube and the T-piece was connected to the endotracheal tube. T-piece ventilation was powered by air entrainment nebulizer, which can deliver FiO_2_ of 0.21–1.00. The air entrainment nebulizer was set at a flow of 8 L/min to provide FiO_2_ of 0.4 (Additional file 1: Fig. S1). In patients assigned to receive the high-flow oxygen ventilation strategy during SBT (high-flow group), the ventilator was disconnected from the endotracheal tube and a high-flow oxygen device (Optiflow; Fisher & Paykel Healthcare) was connected to the endotracheal tube through a specific tracheostomy connection interface (Fisher & Paykel Healthcare) (Additional file 1: Fig. S2). FiO_2_ was set as 0.4, and flow was set at 60 L/min to gain the maximum benefit from high-flow oxygen device [15, 19]. To minimize the effects of oxygenation on the weaning failure, FiO_2_ level was set at 0.4 in both groups [1, 17, 20]. In both groups, SBT was performed for 30–60 min (or less in case of clinical intolerance). Failure of SBT was defined by international guidelines [1] (Additional file 1: Table S1). All patients who successfully completed SBT were protocolized to be reconnected to mechanical ventilation using the previous ventilatory parameters for at least 1 h rest and then directly extubated in both groups [21]. Patients who did not tolerate the SBT were reconnected to mechanical ventilation and received once-daily SBT using the same method according to the assigned group within 72 h after starting the first SBT. Patients who did not complete the SBT successfully within 72 h after the first SBT were classified as weaning failure. According to previous studies, the prophylactic use of HFNC and/or NIV after extubation was considered for all patients for at least 48 h, but was not protocolized and remained at the discretion of ICU attending physician (more details in Additional file 1: Appendix S1) [11–13].

### Outcomes

The primary endpoint was the rate of weaning failure on day 2, which was defined as either the failure of SBT within 72 h after starting the first SBT or the need for reintubation or death within 48 h following extubation [1]. The secondary endpoints included weaning failure on day 7 (defined as either the failure of SBT within 72 h after starting the first SBT or the need for reintubation or death within 7 days following extubation) [2], successful SBT within 72 h after starting the first SBT, extubation after the first SBT, reintubation within 48 h after extubation, reintubation within 7 days after extubation, length of stay (LOS) in the ICU and hospital, ICU mortality, and in-hospital morality. The exploratory endpoints included use of HFNC and NIV within 48 h after extubation, time to the need for HFNC and NIV, and reasons for weaning failure. Although the trial was originally designed to evaluate the outcome of weaning failure on day 2 according to prespecified subgroups, due to lower event rate than planned, we evaluated the outcome of weaning failure on day 7 according to prespecified subgroups (Additional file 1: Appendix S1).

### Statistical analysis

Considering the weaning failure rates of our medical ICU from 2016 to 2018 and a previous study [22], we expected a weaning failure rate of 42% in patients with T-piece SBT and an absolute decrease in weaning failure rate of 27% in patients with high-flow oxygen SBT. Further details are provided in Additional file 1: Appendix S1, Tables S2 and S3. To achieve 85% power to detect this difference, a sample size of 54 patients in each study group was considered to be adequate for a 2-sided test, an alpha level of 5%, and a maximum dropout rate of 10%.

All analyses were conducted according to the intention-to-treat principle. Continuous variables are reported as mean and standard deviation or median and interquartile range (IQR). Categorical variables are reported as frequency and percentage. Between-group differences in baseline characteristics and clinical outcomes were assessed using the Mann–Whitney *U* test or Student’s *t* test for continuous variables and the chi-square test or Fisher’s exact test for categorical variables. Time-to-event outcomes were estimated by the Kaplan–Meier method and compared using the log-rank test. Post hoc analyses were performed for univariable Cox proportional-hazards regression and subgroup analyses using the outcome of weaning failure on day 7 due to lower event rate of primary outcome than planned. A univariable Cox proportional-hazards regression analysis was used to identify independent variables associated with weaning failure on day 7. In addition, among patients with T-piece SBT, we further analyzed the differences in baseline characteristics and extubation strategies (reconnection to ventilator and prophylactic HFNC and/or NIV) that may be associated with SBT and/or extubation failure between our previous cohort and RCT cohort (the population of the present study). The results are presented as hazard ratio (HR) with 95% confidence interval (CI). To assess whether the treatment effects differed between subgroups, a test for interaction was performed. Repeated-measures analysis of variance was used to compare differences in the changes in the physiological variables from baseline to 1 h after extubation. There were no missing values for any of the variables included in the analyses. All analyses were 2-tailed, and *p* values < 0.05 were considered to be significant. All analyses were conducted using IBM SPSS Statistics for Windows (version 25.0; IBM Corp., Armonk, NY, USA).

## Results

### Patient characteristics

**Fig. 1:**
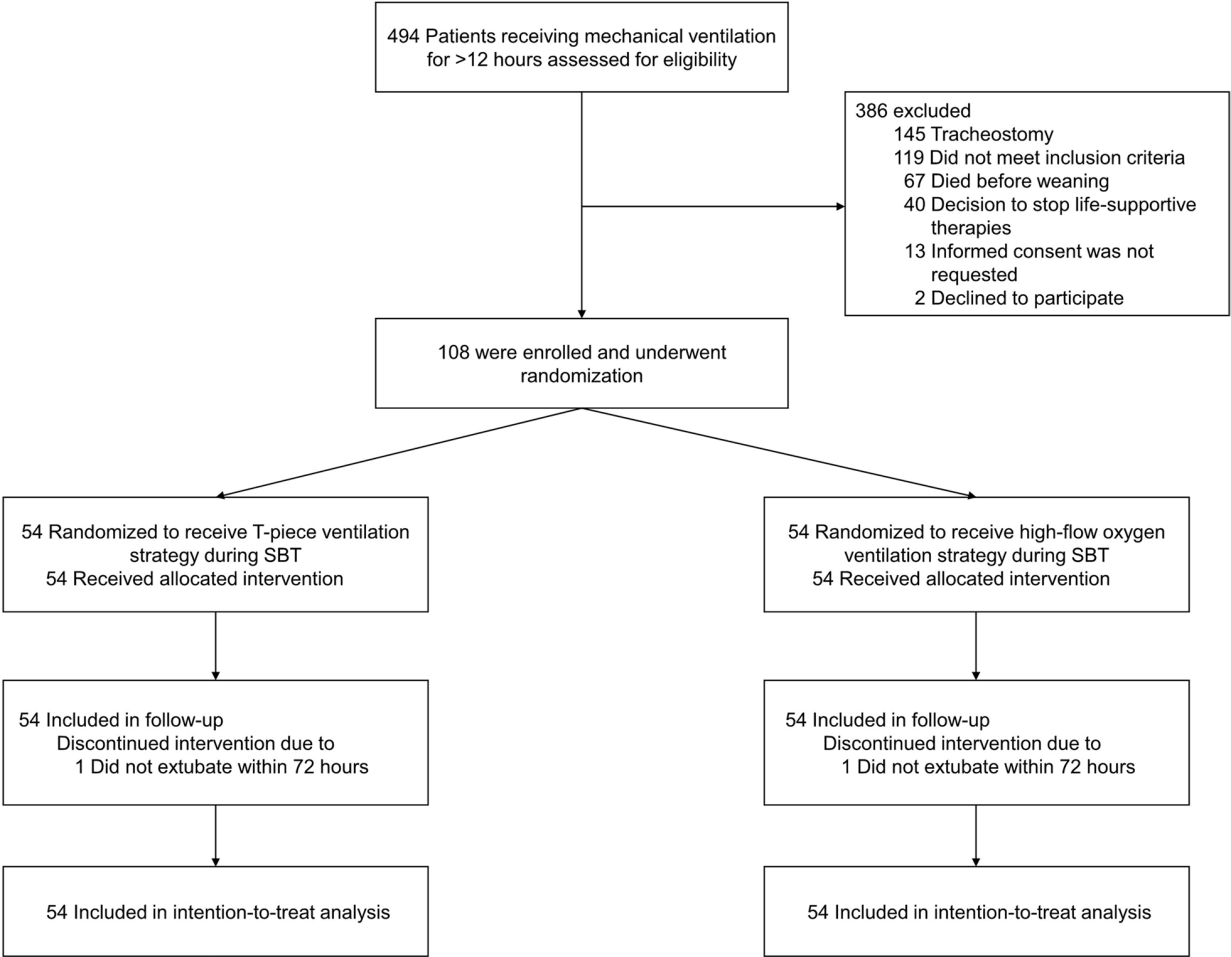
Enrollment, randomization, and follow-up of the study participants. SBT = spontaneous breathing trial

During the study period, a total of 494 patients were assessed for eligibility (Fig. 1), and 108 patients were enrolled and randomly assigned to either the T-piece ventilation strategy (54 patients) or high-flow oxygen ventilation strategy (54 patients) during SBT. There were no dropouts after randomization. The demographic and clinical characteristics of the patients at the baseline did not differ between the two groups (Table 1 and Additional file 1: Table S4). The mean age was 67.0 ± 11.1 years, 64.8% of the patients were men, mean body mass index was 22.3 ± 3.9 kg/m^2^, median duration of mechanical ventilation before the SBT was 4.0 (IQR, 2.6–6.1) days, primary reason for mechanical ventilation was respiratory failure (63.8%), 16% had cardiovascular disease, 26.9% had chronic respiratory disease, and 46.2% were diagnosed with solid malignancy.

**Table 1:** Demographics and clinical characteristics of the patients at the baseline

Characteristics	T-piece SBT (*n* = 54)	High-flow oxygen SBT (*n* = 54)
Age, years	66.3 ± 12.1	67.8 ± 10.0
Male sex	32 (59.3)	38 (70.4)
Body mass index, kg/m^2^	22.9 ± 4.2	21.8 ± 3.6
Length of MV before SBT, days	4.0 (2.6–6.9)	3.9 (2.5–6.0)
APACHE II score at ICU admission	20 ± 7	18 ± 7
Sequential Organ Failure Assessment score	6.2 ± 3.4	6.0 ± 3.9
Reason for intubation		
Respiratory failure	31 (57.4)	38 (70.4)
Nonrespiratory, cardiogenic	10 (18.5)	7 (13.0)
Nonrespiratory, sepsis	7 (13.0)	4 (7.4)
Nonrespiratory, others	6 (11.1)	5 (9.3)
Comorbidity		
Cardiovascular disease	7 (13.0)	10 (18.5)
Chronic respiratory disease	12 (22.2)	17 (31.5)
Diabetes mellitus	18 (33.3)	19 (35.2)
Solid malignancy	26 (48.1)	24 (44.4)
Hematologic malignancy	16 (29.6)	8 (14.8)
Neurologic disease	3 (5.6)	8 (14.8)
Chronic kidney disease	6 (11.1)	7 (13.0)
Chronic liver disease	7 (13.0)	11 (20.4)
Charlson comorbidity index	5.8 ± 2.6	6.4 ± 2.7
Baseline physiological variables		
Arterial blood pH	7.47 ± 0.05	7.46 ± 0.04
PaO_2_/FiO_2_, mm Hg	321 (258–386)	294 (218–390)
PaCO_2_, mm Hg	35.9 ± 5.4	37.6 ± 6.4
Lactate, mmol/L	1.9 (1.4–2.5)	1.4 (1.1–2.2)

Data are reported as *n* (%), mean ± standard deviation, or median (1st–3rd quartile)

*APACHE II* Acute Physiology and Chronic Health Evaluation, *ICU* Intensive care unit, *MV* Mechanical ventilation, *SBT* Spontaneous breathing trial

The results of arterial blood gas analysis and the ventilator settings before the SBT did not differ between the two groups (Table 1 and Additional file 1: Table S4). At the baseline, all patients received pressure support ventilation; the median PaO_2_/FiO_2_ ratio was 307 (IQR, 249–388) mm Hg, the mean PaCO_2_ was 36.7 ± 6.0 mm Hg, and the median P_0.1_ (change in airway pressure during a brief [100 ms] airway occlusion at the initiation of patient inspiratory effort) [23] was 1.3 (IQR, 0.8–1.9) cm H_2_O. The median duration of the first SBT did not differ significantly between the T-piece group (32 [IQR, 30–35] min) and high-flow group (34 [IQR, 31–39] min) (*p* = 0.082).

### Primary endpoint

Weaning failure on day 2, defined as either the failure of SBT within 72 h after starting the first SBT or the need for reintubation or death within 48 h following extubation, occurred in 5 patients (9.3%) in the T-piece group and 3 patients (5.6%) in the high-flow group; this difference was not significant (difference, 3.7% [95% CI, − 6.1–13.6], *p* = 0.713) (Table 2).

**Table 2:** Primary, secondary, and exploratory outcomes

Outcomes	T-piece SBT (*n* = 54)	High-flow oxygen SBT (*n* = 54)	Risk difference (95% CI)	*p* value
*Primary outcome*				
Weaning failure on Day 2, no. (%)^a^	5 (9.3)	3 (5.6)	3.7 (− 6.1–13.6)	0.713
*Secondary and exploratory outcomes*				
Weaning failure on day 7, no (%)^b^	13 (24.1)	7 (13.0)	11.1 (− 3.4–25.6)	0.215
Successful SBT within 72 h, no. (%)	53 (98.1)	54 (100)	− 1.9 (− 5.5–1.7)	> 0.999
Extubation after first SBT, no. (%)	51 (94.4)	53 (98.1)	− 3.7 (− 10.8–3.4)	0.610
Reintubation within 48 h, n/total (%)^c^	4/53 (7.5)	2/53 (3.8)	3.7 (− 5.0–12.5)	0.674
Reintubation within 7 d, n/total (%)^c^	12/53 (22.6)	6/53 (11.3)	11.3 (− 2.8–25.4)	0.196
Apply NIV within 48 h after extubation, no. (%)^c^	11/53 (20.7)	13/53 (24.5)	− 3.8 (− 19.7–12.1)	0.816
Apply HFNC within 48 h after extubation, no. (%)^c^	40/53 (75.5)	42/53 (79.2)	− 3.7 (− 19.7–12.1)	0.816
ICU length of stay, d	3 (1–7)	2 (1–5)		0.107
Hospital length of stay, d	22 (12–44)	25 (16–46)		0.435
ICU mortality, no. (%)	3 (5.6)	3 (5.6)	0.0 (− 8.6–8.6)	> 0.999
Hospital mortality, no. (%)	21 (38.9)	19 (35.2)	3.7 (− 14.5–21.9)	0.842

*HFNC* High-flow nasal cannula, *ICU* Intensive care unit, *NIV* Noninvasive ventilation, *SBT* Spontaneous breathing trial

^a^Defined as either the failure of SBT within 72 h after starting the first SBT or the need for reintubation or death within 48 h following extubation

^b^Defined as either the failure of SBT within 72 h after starting the first SBT or the need for reintubation or death within 7 days following extubation

^c^Among patients extubated after successful SBT within 72 h

### Secondary endpoints

**Fig. 2:**
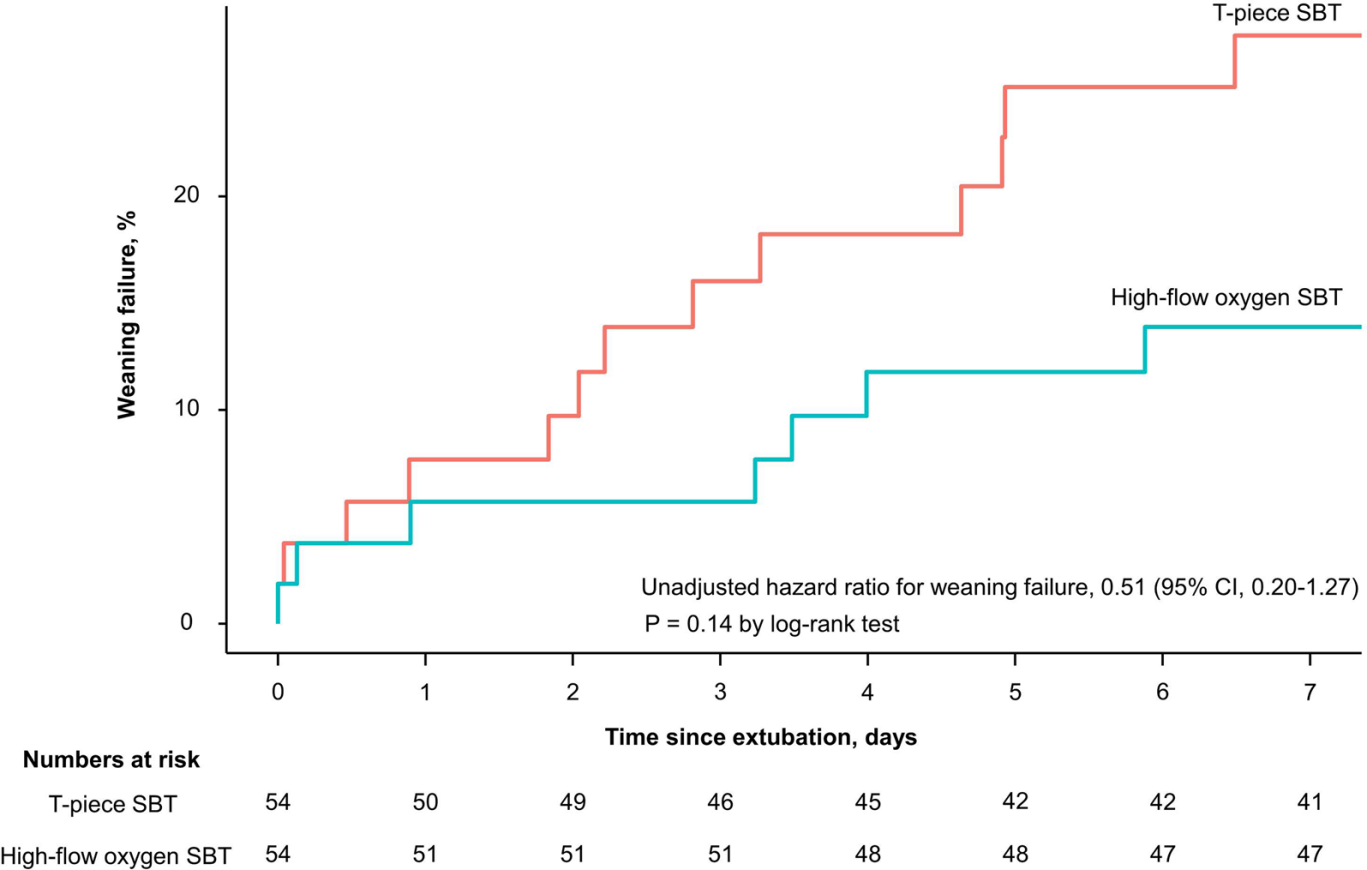
Kaplan–Meier analysis of reintubation within 7 days after extubation in each group. CI = confidence interval; SBT = spontaneous breathing trial

Weaning failure on day 7, defined as either the failure of SBT within 72 h after starting the first SBT or the need for reintubation or death within 7 days following extubation, occurred in 13 patients (24.1%) in the T-piece group and 7 patients (13.0%) in the high-flow group; this difference was not significant (difference, 11.1% [95% CI, − 3.4–25.6], *p* = 0.215) (Table 2). The Kaplan–Meier curves for reintubation within 7 days after extubation are shown in Fig. 2. After starting the first SBT, 53 patients (98.1%) in the T-piece group and 54 patients (100%) in the high-flow group passed the SBT within 72 h (difference, − 1.9% [95% CI, − 5.5–1.7], *p* > 0.999). Of the patients extubated after the SBT, reintubation within 48 h and 7 days occurred in 4 patients (7.5%) and 12 patients (22.6%) in the T-piece group and 2 patients (3.8%) and 6 patients (11.3%) in the high-flow group, respectively. These differences were not significant: The differences were 3.7% (95% CI, − 5.0–12.5, *p* = 0.674) and 11.3% (95% CI, − 2.8–25.4, *p* = 0.196), respectively.

The LOS in the ICU and hospital did not differ between groups. The ICU LOS was 3 (IQR, 1–7) days in the T-piece group and 2 (IQR, 1–5) days in the high-flow group (*p* = 0.107). The hospital LOS was 22 (IQR, 12–44) days in the T-piece group and 25 (IQR, 16–46) days in the high-flow group (*p* = 0.435). The ICU mortality rate was the same in both groups (5.6% or 3 of 54 patients in each group, *p* > 0.999). The hospital mortality rates were 38.9% (21 of 54 patients) in the T-piece group and 35.2% (19 of 54 patients) in the high-flow group (difference, 3.7% [95% CI, − 14.5–21.9], *p* = 0.842).

### Exploratory endpoints

HFNC within 48 h after extubation was applied in 75.5% (40 of 53 patients) in the T-piece group and in 79.2% (43 of 53 patients) in the high-flow group (difference, − 3.7% [95% CI, − 19.7–12.1], *p* = 0.816). NIV within 48 h after extubation was applied in 20.7% (11 of 53 patients) in the T-piece group and 24.5% (13 of 53 patients) in the high-flow group (difference, − 3.8% [95% CI, − 19.7–12.1], *p* = 0.816) (Table 2). The median time to the use of HFNC or NIV after extubation did not differ significantly between the two groups: 1 (IQR, 1–1) min in the T-piece group and 1 (IQR, 1–6) min in the high-flow group (*p* = 0.938). Overall, the median duration of prophylactic use of HFNC or NIV after extubation was 2.0 (IQR, 0.3–5.2) days and did not differ significantly between the two groups: 2.5 (IQR, 0.1–4.0) days in the T-piece group and 1.9 (IQR, 0.7–5.8) days in the high-flow group (*p* = 0.717). More detailed data regarding the settings of prophylactic use of HFNC or NIV and patients’ characteristics associated with prophylactic use of HFNC or NIV after extubation are provided in Additional file 1: Appendix S2 and Table S5.

Among the 8 patients with weaning failure on day 2, inability to clear secretions (3 of 8 patients) was the most common primary reason, followed by persistent postextubation respiratory failure (2 of 8 patients) (Additional file 1: Table S6). Among patients with weaning failure on day 7, inability to clear secretions (7 of 20 patients) was also the most common reason, followed by persistent postextubation respiratory failure (5 of 20 patients) and hemodynamic impairment (3 of 20 patients) (Additional file 1: Table S6). During the study, no adverse events led to withdrawal from the trial, and no serious adverse events were recorded.

### Post hoc analysis

**Fig. 3:**
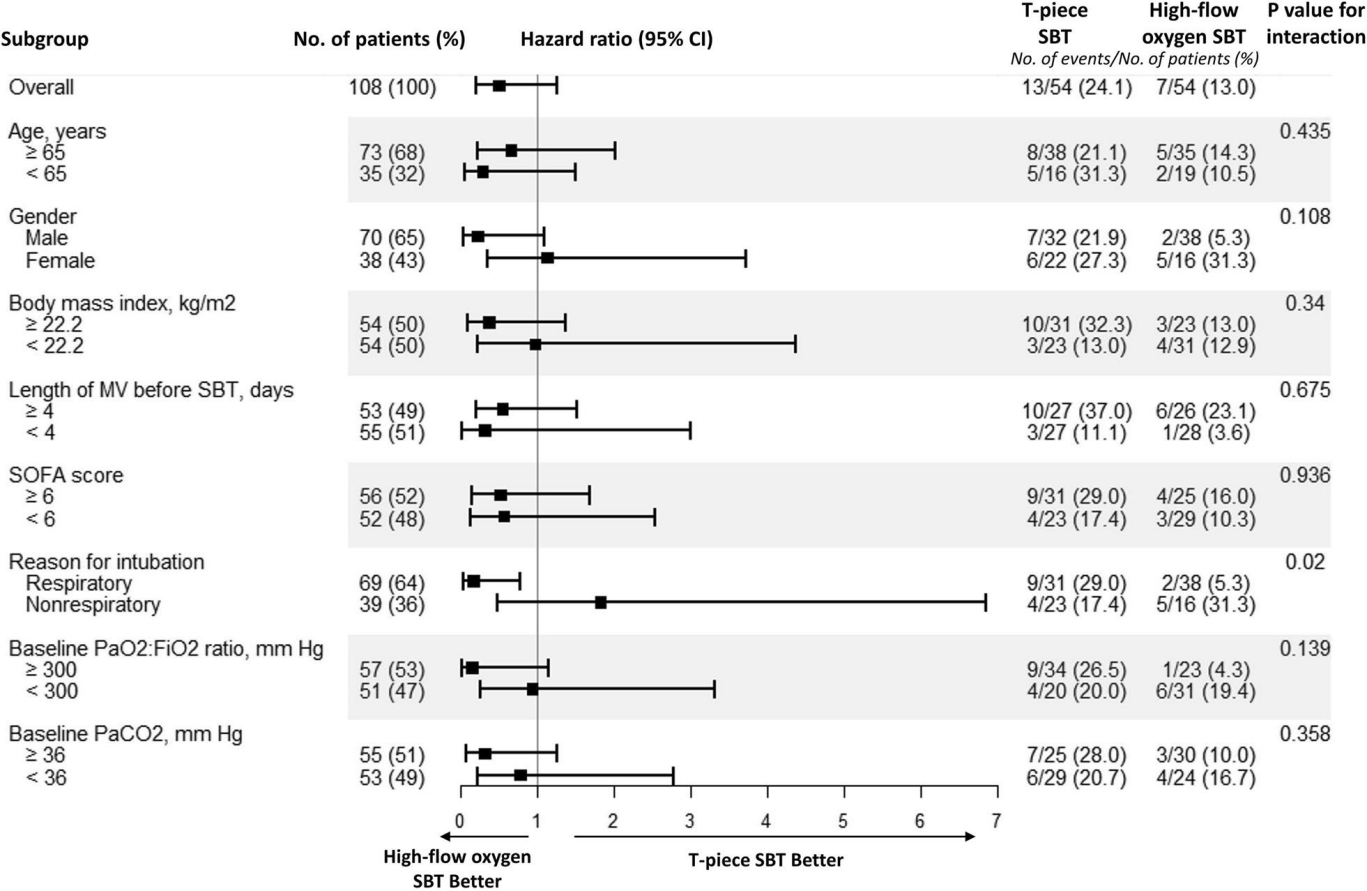
Unadjusted hazard ratios for weaning failure on day 7 in subgroups. MV = mechanical ventilation; SBT = spontaneous breathing trial; and SOFA = Sequential Organ Failure Assessment

The HRs for weaning failure on day 7 in subgroups are shown in Fig. 3. Compared with the group given the T-piece SBT, the HRs for weaning failure on day 7 for the group given high-flow oxygen SBT were 0.17 (95% CI, 0.04–0.78) among patients intubated because of respiratory failure and 1.83 (95% CI, 0.49–6.84) among patients intubated because of nonrespiratory failure (*p* for interaction = 0.020). No significant interactions were found for other subgroup comparisons. A detailed description of the differences in baseline characteristics and extubation strategies between T-piece SBT and high-flow oxygen SBT groups among patients intubated because of respiratory failure is provided in Additional file 1: Appendix S2 and Table S7.

In the univariable Cox analysis, the use of the high-flow oxygen ventilation strategy during SBT (HR, 0.51 [95% CI, 0.20–1.27]) was not significantly associated with weaning failure on day 7. Duration of mechanical ventilation before the SBT (HR, 1.37 [95% CI, 1.21–1.55]) and body mass index (HR, 1.14 [95% CI, 1.04–1.25]) were significantly associated with weaning failure on day 7 (Additional file 1: Table S8). There were no significant between-group differences in all physiological variables from baseline to 1 h after extubation. Additionally, there were no significant interactions in all physiological variables between time and group, indicating no change in the between-group differences over time (Additional file 1: Appendix S2 and Table S9). A detailed description of the differences in baseline characteristics and extubation strategies between our previous and RCT cohorts among patients with T-piece SBT is provided in Additional file 1: Appendix S2, Tables S10 and S11.

## Discussion

In this randomized trial of patients receiving mechanical ventilation, the high-flow oxygen ventilation strategy during SBT did not reduce the risk of weaning failure on days 2 and 7 significantly when compared with the T-piece ventilation strategy during SBT. However, the high-flow oxygen ventilation strategy during SBT was significantly associated with a lower rate of weaning failure on day 7 among those patients intubated because of respiratory failure. The ICU and hospital LOS and mortality rates did not differ significantly between the two groups.

Previous studies have shown inconsistent results regarding which ventilation strategy during SBT is the most appropriate for extubation and liberation from mechanical ventilation [18, 24, 25]. In a post hoc analysis of the HIGH-WEAN trial that included 641 patients at high risk of extubation failure, the initial SBT using PSV was significantly associated with a higher rate of successful extubation compared with the initial SBT using the T-piece [18]. One meta-analysis showed that patients undergoing PSV SBT were more likely to be extubated successfully than those undergoing T-piece SBT (risk ratio, 1.06 [95% CI, 1.02–1.10]; 11 trials, *n* = 1904) [24]. However, a more recent meta-analysis that included 10 RCTs (*n* = 3165) found no significant difference in the successful extubation rate between the T-piece and PSV SBTs (odds ratio, 0.99 [95% CI, 0.78–1.26]) [25]. Thus, further research is warranted to determine the best ventilation strategies during SBT.

One recent pilot RCT of patients at high risk of weaning failure showed that the high-flow oxygen SBT neither accelerated mechanical ventilation weaning nor increased the reintubation rate compared with T-piece SBT [16]. Interestingly, the probability of reintubation over time was significantly higher for T-piece SBT than for high-flow oxygen SBT (*p* = 0.04). However, these results must be interpreted carefully because the prophylactic use of HFNC and/or NIV after extubation was not protocolized and patients with high-flow oxygen SBT were more likely to receive prophylactic use of HFNC (odds ratio, 3.7 [95% CI, 1.3–10.9]). In the present study, the rates of weaning failure on days 2 and 7 did not differ between the T-piece and high-flow oxygen ventilation strategies during SBT. However, our post hoc subgroup analyses showed that patients intubated because of respiratory failure had a significantly lower risk of weaning failure on day 7 with the high-flow oxygen SBT.

There are some plausible explanations for the current findings. First, prolonged mechanical ventilation and comorbidity involving chronic respiratory disease increase the risk of ICU-acquired weakness [26, 27]. The effort induced by SBT may be exhausting for critically ill patients with these risk factors. A previous RCT showed that a 1 h rest after a successful SBT significantly reduced the rates of reintubation and postextubation respiratory failure in critically ill patients [21]. Although another recent RCT showed that a 1 h rest after a successful SBT did not reduce the rate of reintubation, a positive effect for reintubation was observed when the duration of mechanical ventilation was > 72 h before extubation [28]. Moreover, another RCT reported that the use of a shorter, less demanding SBT strategy produced a significantly higher rate of successful extubation [5]. In the present study, patients intubated because of respiratory failure had a longer duration of mechanical ventilation before the SBT (4.1 [2.9–6.2] days vs. 2.8 [2.0–5.6] days, *p* = 0.046) and greater comorbidity involving chronic respiratory disease (36.2% vs. 10.3%, *p* = 0.007) and were, therefore, more likely to have decreased physiological and respiratory reserve. There are several methods for diagnosis of ICU-acquired weakness, such as 6-grade Medical Research Council sum score, electrophysiological studies, and nerve and muscle biopsies [26]. However, our study did not measure these outcomes and need further studies to better understand the underlying pathophysiological mechanisms.

A second possible explanation is that high-flow oxygen therapy has physiological advantages over conventional oxygen therapy, including improved clearance of secretions, decreased inspiratory effort and work of breathing, reduced dead space ventilation, improved lung compliance, provision of a modest positive end-expiratory pressure effect, and improved ventilation and oxygenation through alveolar recruitment [9, 10, 29]. Accordingly, high-flow oxygen SBT may be a less demanding SBT strategy than T-piece SBT and may be beneficial for patients with decreased physiological and respiratory reserve. One previous study showed that HFOT_TRACHEAL_ improved oxygenation compared with T-piece ventilation [30]. However, mean airway pressure was only slightly different (mean difference, + 0.7 cm H_2_O, *p* = 0.01) between HFOT_TRACHEAL_ and T-piece ventilation. In addition, there were no significant differences in other outcomes including end-expiratory lung volume, respiratory rate, heart rate, and subjective dyspnea. Moreover, the physiological effects of HFOT_TRACHEAL_ may differ from HFNC. A recent crossover study showed that a minimum gas flow of 50 L/min HFOT_TRACHEAL_ is needed to limit the inspiratory airway pressure swing, reduce respiratory rate, and improve oxygenation, as compared to standard oxygen [15]. Interestingly, at same gas flow, HFNC produces higher tracheal expiratory pressure than HFOT_TRACHEAL_, suggesting that the physiological effects of HFOT_TRACHEAL_ are milder than HFNC. These findings may be explained by the fact that the HFOT_TRACHEAL_ is open-circuit system and the tracheal oxygen delivery bypasses the larynx and upper airway. Accordingly, all these aforementioned factors may potentially contribute to the lack of differences in the primary outcome between high-flow oxygen SBT and T-piece SBT. A further larger RCT is warranted to confirm these possibilities.

Our study has several limitations. First, our study may have been underpowered to detect a clinically important treatment effect for the comparison of high-flow oxygen SBT versus T-piece SBT, and a higher percentage of patients with simple weaning and a lower weaning failure rate than expected should be considered when interpreting the findings. According to the WIND classification, the percentage of patients with simple weaning in our trial (96%) is higher than percentages observed in our previous cohort (76%) and in previous studies (ranging from 68 to 88%) [2, 5, 18]. In clinical practice, the decision to liberation from mechanical ventilation is made on an individualized basis. Some patients who do not meet all the criteria of weaning readiness or SBT success may be ready for attempts at the liberation from mechanical ventilation and those patients undergo extubation [17]. On the other hand, these attempts can potentially lead to premature extubation which may require reintubation. There are several risk factors associated with SBT and/or extubation failure, including advanced age, hypoxemia, hypercapnia, chronic cardiovascular disease, reason for intubation, and duration of mechanical ventilation [31]. Extubation strategies (reconnection to ventilator and prophylactic HFNC and/or NIV) may significantly lower the rates of reintubation [11–13, 21]. In the present study, all the patients satisfied the criteria of weaning readiness and/or SBT success. Moreover, our strict inclusion criteria might have introduced a selection bias in the study with a high pretest probability of successful weaning and, therefore, could not detect a clinically important treatment effect for the comparison of high-flow oxygen SBT versus T-piece SBT. Although this could mitigate the risk of premature extubation, it could potentially lead to delayed extubation which might contribute to a worse outcome [31]. However, our RCT cohort had similar clinical characteristics, such as age and sequential organ failure assessment (SOFA) score, compared to previous studies, whereas the median duration of mechanical ventilation before the SBT in our RCT cohort was similar to or shorter than that of previous studies [5, 18]. Moreover, the median duration of mechanical ventilation before the SBT did not differ between our previous and RCT cohorts. Compared to our RCT cohort, our previous cohort more frequently had cardiovascular disease, had a lower PaO_2_/FiO_2_ ratio, had a higher PaCO_2_ level, and had a lower compliance rate of extubation strategies. Accordingly, these distinct baseline characteristics, extubation strategies, compliance of the criteria of weaning readiness or SBT success, and percentage of patients with simple weaning would potentially contribute to a lower than expected rate of weaning failure. Ultimately, these findings suggest a need for a fully powered trial to understand the effects of high-flow oxygen SBT on weaning failure.

Second, given the nature of the ventilation strategy during SBT, we could not blind the participants or ICU attending physicians. Third, we included only patients admitted to the medical ICU to ensure a study population as homogeneous as possible, although this may have limited the generalizability of our results. In addition, this study was a single-center RCT, possibly affecting the generalizability of our results. Fourth, the compliance of extubation strategies may significantly affect the rates of reintubation [11–13, 21]. In the present study, all patients who successfully completed the SBT were protocolized to be reconnected to mechanical ventilation for at least 1 h rest and then directly extubated in both groups. Although the decision was left to the discretion of the ICU attending physician, there were no significant differences in the rates and the duration of prophylactic use of HFNC or NIV after extubation between the two groups. Moreover, the median time to the use of HFNC or NIV after extubation did not differ significantly between the two groups. However, there may be unidentified or unmeasured variables that possibly could have influenced the outcome. In addition, the results of our subgroup analysis should be considered exploratory and interpreted with caution, given that the analysis was performed post hoc. Fifth, our study could not provide any information on the effectiveness of high-flow oxygen SBT compared to PSV SBT which is less demanding SBT strategy than T-piece SBT and warrant further studies. Finally, it is currently unavailable to measure esophageal pressure in our country. Therefore, the physiological variables, including pressure time product and work of breathing, could not be measured in the present study. A better understanding of the physiological effects of different SBT strategies will assist in selecting the optimal SBT strategies [4]. Therefore, future study is needed to investigate the physiological effects of different SBT strategies.

## Conclusions

Among patients receiving mechanical ventilation, the high-flow oxygen ventilation strategy during SBT did not significantly reduce the risk of weaning failure compared with the T-piece ventilation strategy during SBT. However, the study may have been underpowered to detect a clinically important treatment effect for the comparison of high-flow oxygen SBT versus T-piece SBT, and a higher percentage of patients with simple weaning and a lower weaning failure rate than expected should be considered when interpreting the findings.

## Supplementary Information

**Additional file 1.** Supplementary material.

## Data Availability

The datasets generated during the current study are available from the corresponding author upon reasonable request.

## Abbreviations

CI: Confidence interval
HFNC: High-flow nasal cannula
HFOT_TRACHEAL_: High-flow oxygen therapy via tracheostomy
HR: Hazard ratio
ICU: Intensive care unit
IQR: Interquartile range
LOS: Length of stay
NIV: Noninvasive ventilation
PSV: Pressure support ventilation
RCT: Randomized controlled trial
SBT: Spontaneous breathing trial
SOFA: Sequential organ failure assessment
