# Supplementary material for: Effect of high-flow oxygen versus T-piece ventilation strategies during spontaneous breathing trials on weaning failure among patients receiving mechanical ventilation: a randomized controlled trial
Source: Crit Care. 2022 Dec 23;26:402. doi: 10.1186/s13054-022-04281-w (PMC9783722; doi:10.1186/s13054-022-04281-w)
Supplement: Supplementary file 1 — Additional file 1. Supplementary material. [file 13054_2022_4281_MOESM1_ESM.pdf]

Additional file 1:

Effect of high-flow oxygen versus T-piece ventilation strategies during spontaneous breathing trials on weaning failure among patients receiving mechanical ventilation: a randomized controlled trial

Hong Yeul Lee[1], Jinwoo Lee[2], Sang-Min Lee[1,2]

Affiliations:

[1]Department of Critical Care Medicine, Seoul National University Hospital, Seoul, Republic of Korea

[2]Division of Pulmonary and Critical Care Medicine, Department of Internal Medicine, Seoul National University Hospital, Seoul National University College of Medicine, Seoul, Republic of Korea

Corresponding author:

Sang-Min Lee, MD, PhD

Professor

Division of Pulmonary and Critical Care Medicine, Department of Internal Medicine, Seoul National University Hospital, Seoul National University College of Medicine, 101 Daehak-ro, Jongno-gu, Seoul 03080, Republic of Korea;

Tel: +82-2-2072-0833;

Fax: +82-2-762-9662;

E-mail: sangmin2@snu.ac.kr

List of contents

Appendix S1. Supplementary methods……………………………………………………………….……………3

Appendix S2. Supplementary results……………………………………………………………………………6

Table S1. Criteria for spontaneous breathing trial failure..……………………………………………………9

Table S2. Demographics and clinical characteristics of the previous cohort at the baseline ………………10

Table S3. Primary, secondary, and exploratory outcomes of the previous cohort……………………………11

Table S4. Demographics and clinical characteristics of the patients at the baseline…………………………12

Table S5. Univariable logistic regression analysis with the dependent variable being the prophylactic use of HFNC or NIV after extubation………………………………………………………………………………13

Table S6. Reasons for weaning failure on Day 2 and Day 7 ………………………..….……………………14

Table S7. Demographics and clinical characteristics of the patients intubated because of respiratory failure……………………………………………………………………………………………….……15

Table S8. Univariable Cox proportional-hazards regression analysis for predicting weaning failure on Day 7……………………………………………………………………...……………….….…………16

Table S9. Changes of physiological variables during the study period…………………….….………………17

Table S10. Comparison of clinical characteristics between the cohorts among patients with T-piece spontaneous breathing trial…………………………………………………………..…………...…..…18

Table S11. Comparison of clinical outcomes between the cohorts among patients with T-piece spontaneous breathing trial…………………………………………………………………………………………...…19

Figure S1. T-piece ventilation strategy during spontaneous breathing trial……………….………….……20

Figure S2. High-flow oxygen ventilation strategy during spontaneous breathing trial…………………..…21

Appendix S1. Supplementary methods

Participants

All consecutive adult patients admitted to the medical ICU who required endotracheal intubation and mechanical ventilation underwent screening before enrollment. Patients who were at least 18 years of age were eligible for inclusion if they were receiving mechanical ventilation for at least 12 h, had recovered from the precipitating illness, and had fulfilled the weaning readiness criteria. Weaning readiness was defined as meeting all of the following criteria according to international guidelines [1, 2]: ratio of arterial oxygen pressure (PaO_2) to fraction of inspired oxygen (FiO_2) ≥150 mm Hg with a positive end-expiratory pressure (PEEP) ≤8 cm H_2O and FiO_2 ≤0.4, arterial pH >7.35, rapid shallow breathing index (defined as the ratio of respiratory rate to tidal volume) <105, maximal inspiratory pressure <–20 cm H_2O, absence of electrocardiographic signs of myocardial ischemia, no vasoactive drugs or stable vital signs with the use of vasoactive drugs, heart rate <140/min, temperature <38°C, hemoglobin concentration >8 g/dL, no need for sedatives or stable mental status with the use of sedatives, presence of respiratory stimulus, appropriate spontaneous cough, and absence of excessive tracheobronchial secretions. The exclusion criteria were tracheostomy and the decision to stop life-supportive therapies.

Interventions

All patients who successfully completed SBT were protocolized to be reconnected to mechanical ventilation using the previous ventilatory parameters for at least 1 hour rest and then directly extubated in both groups. Patients who did not tolerate the SBT were reconnected to mechanical ventilation and received once-daily SBT using the same method according to the assigned group within 72 h after starting the first SBT. Patients who did not complete the SBT successfully within 72 h after the first SBT were classified as weaning failure. The prophylactic use of HFNC and/or NIV after extubation was considered for all patients for at least 48 h, but was not protocolized and remained at the discretion of ICU attending physician. The prophylactic use of NIV was carried out with mechanical ventilator with face mask-delivered NIV pressure-support mode with a positive end-expiratory pressure level between 5–10 cm H_2O, a minimal pressure-support level of 5 cm H_2O targeting a tidal volume around 6–8 mL/kg of predicted body weight, and a FiO_2 adjusted to achieve adequate oxygenation (SpO_2 ≥92%). The prophylactic use of HFNC was carried out with high-flow oxygen device (Optiflow; Fisher & Paykel Healthcare). Flow was initially set at 60 L/min and FiO_2 adjusted to achieve adequate oxygenation (SpO_2 ≥92%).

Outcomes

Although the trial was originally designed to evaluate the outcome of weaning failure on day 2 according to prespecified subgroups, due to lower event rate than planned, we evaluated the outcome of weaning failure on day 7 according to prespecified subgroups. Post-hoc subgroup analyses were performed according to age (<65 years vs. ≥65 years), sex, body mass index (below vs. above the median of 22.2 kg/m^2), length of mechanical ventilation before SBT (below vs. above the median of 4 days), baseline sequential organ failure assessment (SOFA) score (below vs. above the median of 6), reason for intubation (respiratory vs. nonrespiratory), baseline PaO_2:FiO_2 ratio (<300 mm Hg vs. ≥300 mm Hg), and baseline $PaCO_2$ level (below vs. above the median of 36 mm Hg).

Sample size

The rates of weaning failure during T-piece SBT were varied widely from 14.6% to 44.0% across the recent randomized controlled trials which were published after 2015 [3-8]. There are several risk factors associated with SBT and/or extubation failure, including advanced age, hypoxemia, hypercapnia, chronic cardiovascular disease, chronic respiratory disease, reason for intubation, duration of mechanical ventilation, and baseline severity of illness [9]. Among the aforementioned RCTs, trials with low weaning failure rates tended to include patients who are younger, have higher non-respiratory reasons for intubation, lower severity scores, or fewer comorbidities than those with high weaning failure rate.

When we planned the study in 2018 and 2019, there was no published study that investigated the efficacy and safety of the high-flow oxygen SBT. Therefore, we considered the weaning failure rates in our medical ICU at Seoul National University Hospital from 2016 to 2018. During the 2016 to 2018, a total of 312 patients were weaned from mechanical ventilation; of these, 194 (62.2%) used T-piece, 66 (21.2%) used continuous positive airway pressure, 31 (9.9%) used pressure support ventilation, and 21 (6.7%) used high-flow oxygen ventilation during SBT. In our previous cohort, the demographic and clinical characteristics of the patients at the baseline did not differ between the T-piece SBT and high-flow oxygen SBT groups (Additional file 1: Table S2). The mean age was 66.4 ± 14.2 years, 65.6% of the patients were men, mean body mass index was 22.3 ± 3.9 kg/m^2, median duration of mechanical ventilation before the SBT was 3.8 (IQR, 2.2–6.5) days, and primary reason for mechanical ventilation was respiratory failure (60.9%), which were similar to our RCT cohort (the population

of the present study). Weaning failure on day 2 occurred in 75 patients (38.7%) in the T-piece group and 3 patients (14.3%) in the high-flow group (p = 0.049) (Additional file 1: Table S3). Successful SBT within 72 h occurred in 178 patients (91.8%) in the T-piece group and 21 patients (100%) in the high-flow group (p = 0.352). HFNC or NIV within 48 h after extubation was applied in 56.7% (101 of 178 patients) in the T-piece group and in 90.5% (19 of 21 patients) in the high-flow group (p = 0.006). Among patients who successfully completed the SBT, 31.5% (56 of 178 patients) in the T-piece group and 57.1% (12 of 21 patients) in the high-flow group were reconnected to mechanical ventilation before extubation (p = 0.035). Based on the results from our previous cohort, we assumed a weaning failure rate of 15% in patients with high-flow oxygen SBT. In addition, we selected the study with the most similar baseline characteristics and weaning failure rate of T-piece SBT from our previous cohort, and based on this, we assumed a weaning failure rate of 42% in patients with T-piece SBT [10].

Appendix S2. Supplementary results

Exploratory endpoints

Overall, the median duration of prophylactic use of HFNC or NIV after extubation was 2.0 (IQR, 0.3–5.2) days and did not differ significantly between the two groups: 2.5 (IQR, 0.1–4.0) days in the T-piece group and 1.9 (IQR, 0.7–5.8) days in the high-flow group (p = 0.717). The median time to the use of HFNC or NIV after extubation did not differ significantly between the two groups: 1 (IQR, 1–1) min in the T-piece group and 1 (IQR, 1–6) min in the high-flow group (p = 0.938). The median settings of prophylactic HFNC and NIV at 1 h after extubation were as follows: in patients with prophylactic use of HFNC, the flow rate was 60 (IQR, 60–60) L/min with FiO_2 of 0.40 (IQR, 0.40–0.40); in patients with prophylactic use of NIV, the positive end-expiratory pressure level was 6 (IQR, 5–6) cm H_2O, pressure-support level was 7 (IQR, 6–7) cm H_2O, and FiO_2 was 0.38 (0.30–0.40), resulting in a tidal volume of 8.3 (IQR, 6.8–9.4) mL/kg of predicted body weight.

During the study period, 83 patients underwent prophylactic of HFNC or NIV after extubation and 23 patients did not undergo prophylactic of HFNC or NIV after extubation. There were no significant differences in baseline characteristics between patients with and without prophylactic use of HFNC or NIV after extubation. The median duration of mechanical ventilation before the SBT was numerically shorter in patients without prophylactic use (2.9 [IQR, 1.8–5.2] days) than those with prophylactic use of HFNC or NIV (4.0 [IQR, 2.8–6.1] days) (p = 0.083). At the end of SBT, the median PaO_2:FiO_2 ratio was higher in patients without prophylactic use (360 [IQR, 279–442] mm Hg) than those with prophylactic use of HFNC or NIV (285 [IQR, 212–390] mm Hg) (p = 0.030). In order to determine if there was imbalance between the T-piece and high-flow groups regarding the prophylactic use of HFNC or NIV, we conducted univariable logistic regression analysis with the dependent variable being the prophylactic use of HFNC or NIV after extubation. In the logistic regression analysis, the use of the high-flow oxygen ventilation strategy during SBT was not significantly associated with prophylactic use of HFNC or NIV after extubation (OR, 1.12 [95% CI, 0.44–2.85]). Only the PaO_2:FiO_2 ratio at the end of SBT was significantly associated with prophylactic use of HFNC or NIV after extubation (OR, 0.99 [95% CI, 0.99–1.00]) (Additional file 1: Table S5).

Post-hoc analysis

We further analyzed the differences in baseline characteristics and extubation strategies between T-piece SBT and

high-flow oxygen SBT groups among patients intubated because of respiratory failure. There were no significant differences in baseline characteristics between the two groups, except in the sex, comorbidity with chronic respiratory failure, and serum lactate level (Additional file 1: Table S7). Compared to patients with T-piece SBT, patients with high-flow oxygen SBT were more likely to be men (p = 0.016), more frequently had chronic respiratory failure (p = 0.031), and had a lower serum lactate level (p = 0.015). HFNC within 48 h after extubation was applied in 90.3% (28 of 31 patients) in the T-piece group and in 78.4% (29 of 38 patients) in the high-flow group (p = 0.317). NIV within 48 h after extubation was applied in 32.3% (10 of 31 patients) in the T-piece group and 24.3% (9 of 38 patients) in the high-flow group (p = 0.649). Overall, the median duration of prophylactic use of HFNC or NIV after extubation was 2.9 (IQR, 0.9–5.9) days and did not differ significantly between the two groups: 3.0 (IQR, 1.9–4.8) days in the T-piece group and 1.9 (IQR, 0.7–6.9) days in the high-flow group (p = 0.590). The median time to the use of HFNC or NIV after extubation did not differ significantly between the two groups: 1 (IQR, 1–1) min in the T-piece group and 1 (IQR, 1–1) min in the high-flow group (p = 0.782). The median settings of prophylactic HFNC and NIV at 1 h after extubation were as follows: in patients with prophylactic use of HFNC, the flow rate was 60 (IQR, 60–60) L/min with FiO_2 of 0.40 (IQR, 0.40–0.40); in patients with prophylactic use of NIV, the positive end-expiratory pressure level was 6 (IQR, 5–6) cm H_2O, pressure-support level was 7 (IQR, 6–7) cm H_2O, and FiO_2 was 0.38 (IQR, 0.35–0.43), resulting in a tidal volume of 8.2 (IQR, 6.8–9.4) mL/kg of predicted body weight.

Repeated-measures analysis of variance was used to compare differences in the changes in the physiological variables from baseline to 1 h after extubation. The physiological variables included in this analysis are as follows: arterial blood pH, PaO_2, PaO_2:FiO_2 ratio, $PaCO_2$, bicarbonate, SaO_2, FiO_2, lactate, mean blood pressure, heart rate, and respiratory rate. There were no significant between-group differences in all physiological variables from baseline to 1 h after extubation. Additionally, there were no significant interactions in all physiological variables between time and group, indicating no change in the between-group differences over time (Additional file 1: Table S8).

Post-hoc analysis was performed for the differences in baseline characteristics and extubation strategies that may be associated with SBT and/or extubation failure between our previous and RCT cohorts. In the post-hoc analysis of 248 patients with T-piece SBT, 194 (78.2%) were included in the previous cohort and 54 (21.8%) were included in the RCT cohort. The demographic and clinical characteristics of the patients at the baseline are shown in Additional file 1: Table S9. Compared to the RCT cohort, the previous cohort more frequently had

cardiovascular disease and neurologic disease, had a lower $PaO_2:FiO_2$ ratio, and had a higher $PaCO_2$ level. However, age, body mass index, Acute Physiology and Chronic Health Evaluation (APACHE) II score at admission, Sequential Organ Failure Assessment (SOFA), and median duration of mechanical ventilation before the SBT did not differ between the cohorts. HFNC or NIV within 48 h after extubation was applied in 56.7% (101 of 178 patients) in the previous cohort and in 77.3% (41 of 53 patients) in the RCT cohort (p = 0.011). Reconnection to ventilator after a successful SBT was performed in 31.5% (56 of 178 patients) in the previous cohort and 100% (53 of 53 patients) in the RCT cohort ($p < 0.001$) (Additional file 1: Table S10). The ICU and hospital LOS, and mortality rates did not differ significantly between the cohorts.

Table S1. Criteria for spontaneous breathing trial failure

A failed SBT will be defined by the presence of any ONE of:
(1) PaO_2 < 60 mmHg or SpO_2 < 90% on FiO_2 > 0.5
(2) $PaCO_2$ > 50 mmHg (except for those who have chronic respiratory acidosis)
(3) pH < 7.32
(4) Respiratory rate > 35 /min
(5) Heart rate > 140 /min or cardiac arrhythmia
(6) Systolic blood pressure > 180 mm Hg or < 90 mm Hg
(7) Agitation, anxiety, depressed mental status, diaphoresis, cyanosis
(8) Evidence of increasing respiratory effort
(9) Increased accessory muscle activity
(10) Facial signs of distress, dyspnea

SBT = spontaneous breathing trial

Table S2. Demographics and clinical characteristics of the previous cohort at the baseline

**Characteristics**	**T-piece SBT (n = 194)**	**High-flow SBT (n = 21)**	***p* value**
Age, years	66.5 ± 14.1	64.7 ± 14.7	0.575
Male sex	129 (66.5)	12 (57.1)	0.538
Body mass index, kg/m^2	22.4 ± 3.8	21.7 ± 4.9	0.403
Length of MV before SBT, days	3.8 (2.2–6.1)	5.8 (2.4–8.2)	0.239
APACHE II score at ICU admission	22 ± 8	23 ± 9	0.726
Sequential Organ Failure Assessment score	6.1 ± 3.6	6.0 ± 4.8	0.899
Reason for intubation			0.203
Respiratory failure	117 (60.3)	14 (66.7)	
Nonrespiratory, cardiogenic	24 (12.4)	0 (0)	
Nonrespiratory, sepsis	7 (4.6)	0 (0)	
Nonrespiratory, others	44 (22.7)	7 (33.3)	
Comorbidity			
Cardiovascular disease	77 (39.7)	8 (38.1)	>0.999
Chronic respiratory disease	56 (28.9)	8 (38.1)	0.530
Diabetes mellitus	54 (27.8)	2 (9.5)	0.120
Solid malignancy	51 (26.3)	4 (19.0)	0.646
Hematologic malignancy	33 (17.0)	6 (28.6)	0.313
Neurologic disease	37 (19.1)	2 (9.5)	0.435
Chronic kidney disease	64 (33.0)	3 (14.3)	0.131
Chronic liver disease	30 (15.5)	4 (19.0)	0.910
Baseline physiological variables			
Arterial blood pH	7.44 ± 0.05	7.45 ± 0.05	0.470
PaO_2:FiO_2, mm Hg	289 (226–371)	269 (251–429)	0.619
$PaCO_2$, mm Hg	37.8 ± 7.8	38.2 ± 7.5	0.801
Lactate, mmol/L	1.5 (1.1–2.2)	1.9 (1.2–2.3)	0.416
Maximal inspiratory pressure, mm Hg	32 (25–45)	36 (28–42)	0.961
Rapid shallow breathing index	46 (32–66)	48 (42–66)	0.453

Data are reported as *n* (%), mean ± standard deviation, or median (1st–3rd quartile).

APACHE II = Acute Physiology and Chronic Health Evaluation; ICU = intensive care unit; MV = mechanical ventilation; SBT = spontaneous breathing trial.

Table S3. Primary, secondary, and exploratory outcomes of the previous cohort

**Outcomes**	**T-piece SBT (n = 194)**	**High-flow SBT (n = 21)**	***p* value**
**Primary outcome**			
Weaning failure at Day 2, no. (%)[a]	75 (38.7)	3 (14.3)	0.049
**Secondary and exploratory outcomes**			
Successful SBT within 72 h, no. (%)	178 (91.8)	21 (100)	0.352
Apply NIV or HFNC within 48 hours after extubation, no. (%)[b]	101/178 (56.7)	19/21 (90.5)	0.006
Apply NIV within 48 hours after extubation, no. (%)[b]	20/178 (11.2)	1/21 (4.8)	0.591
Apply HFNC within 48 hours after extubation, no. (%)[b]	92/178 (51.7)	19/21 (90.5)	0.002
ICU length of stay, d	3 (1–6)	3 (2–7)	0.903
Hospital length of stay, d	23 (11–44)	27 (21–54)	0.156
ICU mortality, no. (%)	24 (12.4)	3 (14.3)	>0.999
28-Day mortality, no. (%)	41 (21.1)	5 (23.8)	0.997
Hospital mortality, no. (%)	70 (36.1)	9 (42.9)	0.709
Reconnection to ventilator after a successful SBT[b]	56/178 (31.5)	12/21 (57.1)	0.035

HFNC = high-flow nasal cannula; ICU = intensive care unit; NIV = non-invasive ventilation; SBT = spontaneous breathing trial.

[a] Defined as either the failure of SBT within 72 h after starting the first SBT or the need for reintubation or death within 48 h following extubation

[b] Among patients extubated after successful SBT within 72 hours.

Table S4. Demographics and clinical characteristics of the patients at the baseline

**Characteristics**	**T-piece SBT (n = 54)**	**High-flow oxygen SBT (n = 54)**
Maximal inspiratory pressure, mm Hg	38 (30–48)	40 (30–50)
Rapid shallow breathing index	43 (30–62)	40 (29–54)
Baseline physiological variables		
Arterial blood pH	7.47 ± 0.05	7.46 ± 0.04
PaO_2:FiO_2, mm Hg	321 (258–386)	294 (218–390)
$PaCO_2$, mm Hg	35.9 ± 5.4	37.6 ± 6.4
Lactate, mmol/L	1.9 (1.4–2.5)	1.4 (1.1–2.2)
Mean blood pressure, mm Hg	84 ± 12	88 ± 14
Heart rate, beats/min	83 ± 17	81 ± 16
Body temperature, °C	36.9 ± 0.6	36.8 ± 0.5
Respiratory rate, breaths/min	17 ± 4	17 ± 4
Ventilator settings before SBT		
Pressure-support ventilation	54 (100)	54 (100)
Airway occlusion pressure ($P_{0.1}$), cm H_2O	1.4 (0.8–2.2)	1.2 (0.8–1.8)

Data are reported as *n* (%), mean ± standard deviation, or median (1st–3rd quartile).

SBT = spontaneous breathing trial.

Table S5. Univariable logistic regression analysis with the dependent variable being the prophylactic use of HFNC or NIV after extubation

**Variable**	**Univariable analysis**	**P value**
	**OR (95% CI)**	
High-flow SBT (vs T-piece SBT)	1.12 (0.44–2.85)	0.814
Gender, female	1.69 (0.63–5.09)	0.319
Age, years	1.00 (0.96–1.04)	0.929
Body-mass index, kg/m^2	1.07 (0.95–1.23)	0.288
Length of MV before SBT, days	1.15 (0.96–1.42)	0.153
SOFA score	0.98 (0.86–1.11)	0.698
Cardiovascular disease	0.88 (0.27–3.41)	0.842
Cancer	0.47 (0.14–1.30)	0.168
Charlson comorbidity index	1.06 (0.89–1.28)	0.502
PaO_2:FiO_2 at baseline, mm Hg	1.00 (0.99–1.00)	0.055
PaO_2:FiO_2 at the end of SBT, mm Hg	0.99 (0.99–1.00)	0.023
$PaCO_2$ at baseline, mm Hg	1.06 (0.98–1.16)	0.148
Reason for intubation (respiratory failure)	2.39 (0.93–6.22)	0.069
Chronic respiratory disease	0.71 (0.27–1.90)	0.482

HFNC = high-flow nasal cannula; NIV = non-invasive ventilation; OR = hazard ratio; MV = mechanical ventilation; SBT = spontaneous breathing trial; SOFA = Sequential Organ Failure Assessment

Table S6. Reasons for weaning failure on Day 2 and Day 7

(A) On Day 2

**Variable**	**Total (n = 8)**	**T-piece SBT (n = 5)**	**High-flow oxygen SBT (n = 3)**
Inability to clear secretions	3	1	2
Persistent postextubation respiratory failure	2	2	0
Hemodynamic impairment	1	1	0
Cardiorespiratory arrest	0	0	0
Did not extubated	2	1	1
Died within 48 hours after extubation	0	0	0

(B) On Day 7

**Variable**	**Total (n = 20)**	**T-piece SBT (n = 13)**	**High-flow SBT (n = 7)**
Inability to clear secretions	7	3	4
Persistent postextubation respiratory failure	5	5	0
Hemodynamic impairment	3	1	2
Cardiorespiratory arrest	1	1	0
Did not extubated	2	1	1
Died within 7 days after extubation	2	2	0

Table S7. Demographics and clinical characteristics of the patients intubated because of respiratory failure

**Characteristics**	**T-piece SBT (n = 31)**	**High-flow SBT (n = 38)**	***p* value**
Age, years	65.9 ± 12.1	68.2 ± 10.0	0.387
Male sex	17 (54.8)	32 (84.2)	0.016
Body mass index, kg/m^2	21.8 ± 3.6	21.6 ± 3.6	0.857
Length of MV before SBT, days	4.1 (3.4–6.6)	4.0 (2.8–6.2)	0.437
APACHE II score at ICU admission	18.5 ± 6.4	18.9 ± 6.6	0.839
Sequential Organ Failure Assessment score	5.2 ± 3.5	5.0 ± 2.5	0.770
Reason for intubation			>0.999
Respiratory failure	31 (100)	38 (100)	
Comorbidity			
Cardiovascular disease	3 (9.7)	6 (15.8)	0.696
Chronic respiratory disease	9 (29.0)	22 (57.9)	0.031
Diabetes mellitus	7 (22.6)	12 (31.6)	0.575
Solid malignancy	16 (51.6)	16 (42.1)	0.586
Hematologic malignancy	9 (29.0)	7 (18.4)	0.452
Neurologic disease	3 (9.7)	6 (15.8)	0.696
Chronic kidney disease	2 (6.5)	5 (13.2)	0.605
Chronic liver disease	2 (6.5)	4 (10.5)	0.867
Charlson comorbidity index	5.7 ± 2.6	6.2 ± 2.6	0.458
Baseline physiological variables			
Arterial blood pH	7.47 ± 0.06	7.45 ± 0.04	0.155
PaO_2:FiO_2, mm Hg	306 (251–426)	286 (208–364)	0.311
$PaCO_2$, mm Hg	36.0 ± 6.1	37.6 ± 6.6	0.298
Lactate, mmol/L	1.9 (1.6–2.6)	1.3 (1.0–2.0)	0.015

Data are reported as *n* (%), mean ± standard deviation, or median (1st–3rd quartile).

APACHE II = Acute Physiology and Chronic Health Evaluation; ICU = intensive care unit; MV = mechanical ventilation; SBT = spontaneous breathing trial.

Table S8. Univariable Cox proportional-hazards regression analysis for predicting weaning failure on Day 7

Variable	Univariable analysis	P value
	HR (95% CI)	
High-flow SBT (vs T-piece SBT)	0.51 (0.20–1.27)	0.148
Gender, female	2.40 (0.99–5.79)	0.052
Age, years	0.98 (0.95–1.02)	0.398
Body-mass index, kg/m^2	1.14 (1.04–1.25)	0.004
Length of MV before SBT, days	1.37 (1.21–1.55)	<0.001
SOFA score	1.10 (0.99–1.23)	0.079
Cardiovascular disease	0.53 (0.12–2.29)	0.398
Cancer	0.62 (0.26–1.50)	0.288
Charlson comorbidity index	1.00 (0.85–1.19)	0.970
PaO_2:FiO_2 at baseline, mm Hg	1.00 (0.99–1.00)	0.835
$PaCO_2$ at baseline, mm Hg	1.01 (0.94–1.08)	0.893
Reason for intubation (respiratory failure)	0.68 (0.28–1.63)	0.384
Chronic respiratory disease	0.46 (0.13–1.57)	0.216

HR = hazard ratio; MV = mechanical ventilation; SBT = spontaneous breathing trial; SOFA = Sequential Organ Failure Assessment.

Table S9. Changes of physiological variables during the study period

Variable	Group	Baseline	End of first SBT	1 hour after extubation	*p* value[a]	*p* value for interaction
Arterial blood pH	T-piece SBT	7.47 ± 0.05	7.46 ± 0.06	7.47 ± 0.05	0.600	0.474
	High-flow SBT	7.46 ± 0.04	7.46 ± 0.05	7.46 ± 0.04		
PaO_2, mm Hg	T-piece SBT	100 ± 28	124 ± 38	119 ± 41	0.680	0.294
	High-flow SBT	95 ± 27	128 ± 48	112 ± 41		
PaO_2:FiO_2, mm Hg	T-piece SBT	331 ± 99	311 ± 95	299 ± 101	0.823	0.599
	High-flow SBT	319 ± 126	318 ± 120	288 ± 124		
$PaCO_2$, mm Hg	T-piece SBT	36 ± 5	37 ± 6	35 ± 6	0.171	0.547
	High-flow SBT	38 ± 6	38 ± 6	37 ± 6		
Bicarbonate, mmol/L	T-piece SBT	27 ± 4	26 ± 4	26 ± 4	0.457	0.197
	High-flow SBT	27 ± 4	27 ± 4	27 ± 4		
SaO_2	T-piece SBT	97 ± 2	98 ± 2	97 ± 3	0.244	0.646
	High-flow SBT	96 ± 3	97 ± 2	97 ± 3		
FiO_2	T-piece SBT	31 ± 5	40 ± 0	40 ± 5	0.666	0.997
	High-flow SBT	32 ± 7	40 ± 0	40 ± 6		
Lactate, mmol/L	T-piece SBT	2.2 ± 1.5	2.3 ± 1.6	2.3 ± 1.5	0.062	0.893
	High-flow SBT	1.8 ± 0.9	1.9 ± 0.8	1.9 ± 0.9		
Mean blood pressure, mm Hg	T-piece SBT	84 ± 12	88 ± 14	87 ± 15	0.054	0.956
	High-flow SBT	88 ± 14	92 ± 13	91 ± 18		
Heart rate, beats/min	T-piece SBT	83 ± 17	85 ± 17	85 ± 16	0.575	0.420
	High-flow SBT	81 ± 16	83 ± 17	85 ± 19		
Respiratory rate, breaths/min	T-piece SBT	17 ± 4	18 ± 5	19 ± 6	0.951	0.539
	High-flow SBT	17 ± 4	18 ± 5	19 ± 5		

SBT = spontaneous breathing trial.

[a] *p* values for between-group difference were calculated by repeated-measures analysis of variance.

Table S10. Comparison of clinical characteristics between the cohorts among patients with T-piece spontaneous breathing trial

**Characteristics**	**Previous cohort (n = 194)**	**RCT cohort (n = 54)**	***p* value**
Age, years	66.5 ± 14.1	66.3 ± 12.1	0.893
Male sex	129 (66.5)	32 (59.3)	0.410
Body mass index, kg/m^2	22.4 ± 3.8	22.9 ± 4.2	0.475
Length of MV before SBT, days	3.8 (2.2–6.1)	4.0 (2.6–6.9)	0.549
APACHE II score at ICU admission	22 ± 8	20 ± 7	0.083
Sequential Organ Failure Assessment score	6.1 ± 3.6	6.2 ± 3.4	0.771
Reason for intubation			0.036
Respiratory failure	117 (60.3)	14 (66.7)	
Nonrespiratory, cardiogenic	24 (12.4)	0 (0)	
Nonrespiratory, sepsis	7 (4.6)	0 (0)	
Nonrespiratory, others	44 (22.7)	7 (33.3)	
Comorbidity			
Cardiovascular disease	77 (39.7)	7 (13.0)	<0.001
Chronic respiratory disease	56 (28.9)	12 (22.2)	0.426
Diabetes mellitus	54 (27.8)	18 (33.3)	0.537
Solid malignancy	51 (26.3)	26 (48.1)	0.004
Hematologic malignancy	33 (17.0)	16 (29.6)	0.062
Neurologic disease	37 (19.1)	3 (5.6)	0.029
Chronic kidney disease	64 (33.0)	6 (11.1)	0.003
Chronic liver disease	30 (15.5)	7 (13.0)	0.810
Baseline physiological variables			
Arterial blood pH	7.44 ± 0.05	7.47 ± 0.05	0.003
$PaO_2:FiO_2$, mm Hg	289 (226–371)	321 (258–386)	0.043
$PaCO_2$, mm Hg	37.8 ± 7.8	35.9 ± 5.4	0.042
Lactate, mmol/L	1.5 (1.1–2.2)	1.9 (1.4–2.5)	0.021
Maximal inspiratory pressure, mm Hg	32 (25–45)	38 (30–48)	0.157
Rapid shallow breathing index	46 (32–66)	43 (30–62)	0.162

Data are reported as *n* (%), mean ± standard deviation, or median (1st–3rd quartile).

APACHE II = Acute Physiology and Chronic Health Evaluation; ICU = intensive care unit; MV = mechanical ventilation; RCT = randomized controlled trial; SBT = spontaneous breathing trial.

Table S11. Comparison of clinical outcomes between the cohorts among patients with T-piece spontaneous breathing trial

**Outcomes**	**Previous cohort (n = 194)**	**RCT cohort (n = 54)**	***p* value**
**Primary outcome**			
Weaning failure at Day 2, no. (%)[a]	75 (38.7)	5 (9.3)	0.001
**Secondary and exploratory outcomes**			
Successful SBT within 72 h, no. (%)	178 (91.8)	53 (98.1)	0.180
Apply NIV or HFNC within 48 hours after extubation, no. (%)[b]	101/178 (56.7)	41/53 (77.3)	0.011
Apply NIV within 48 hours after extubation, no. (%)[b]	20/178 (11.2)	11/53 (20.7)	0.120
Apply HFNC within 48 hours after extubation, no. (%)[b]	92/178 (51.7)	40/53 (75.5)	0.004
ICU length of stay, d	3 (1–6)	3 (1-7)	0.692
Hospital length of stay, d	23 (11–44)	22 (12–44)	0.844
ICU mortality, no. (%)	24 (12.4)	3 (5.6)	0.240
28-Day mortality, no. (%)	41 (21.1)	15 (27.8)	0.396
Hospital mortality, no. (%)	70 (36.1)	21 (38.9)	0.827
Reconnection to ventilator after a successful SBT[b]	56/178 (31.5)	53/53 (100)	<0.001

HFNC = high-flow nasal cannula; ICU = intensive care unit; NIV = non-invasive ventilation; RCT = randomized controlled trial; SBT = spontaneous breathing trial.

[a] Defined as either the failure of SBT within 72 h after starting the first SBT or the need for reintubation or death within 48 h following extubation

[b] Among patients extubated after successful SBT within 72 hours.

Figure S1. T-piece ventilation strategy during spontaneous breathing trial

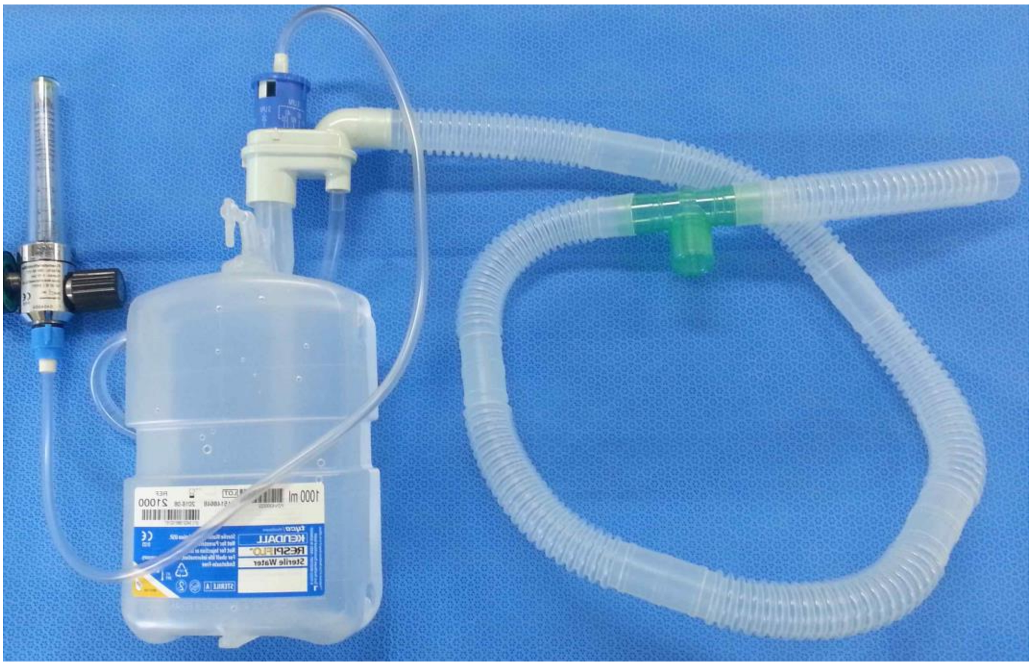

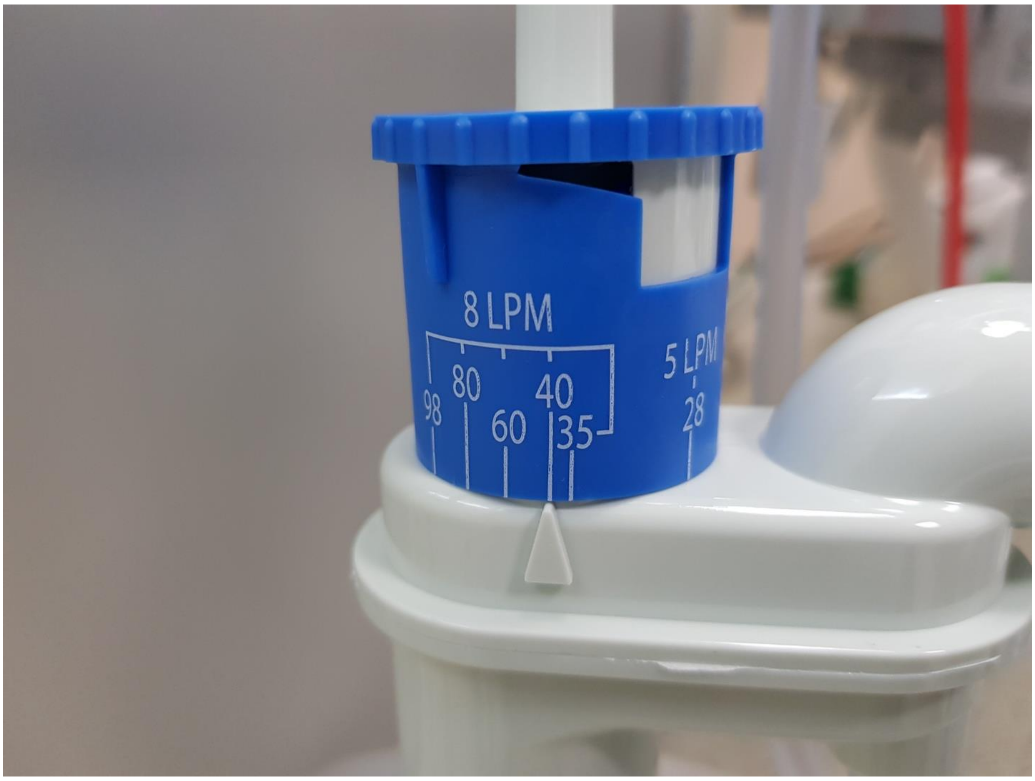

Figure S2. High-flow oxygen ventilation strategy during spontaneous breathing trial

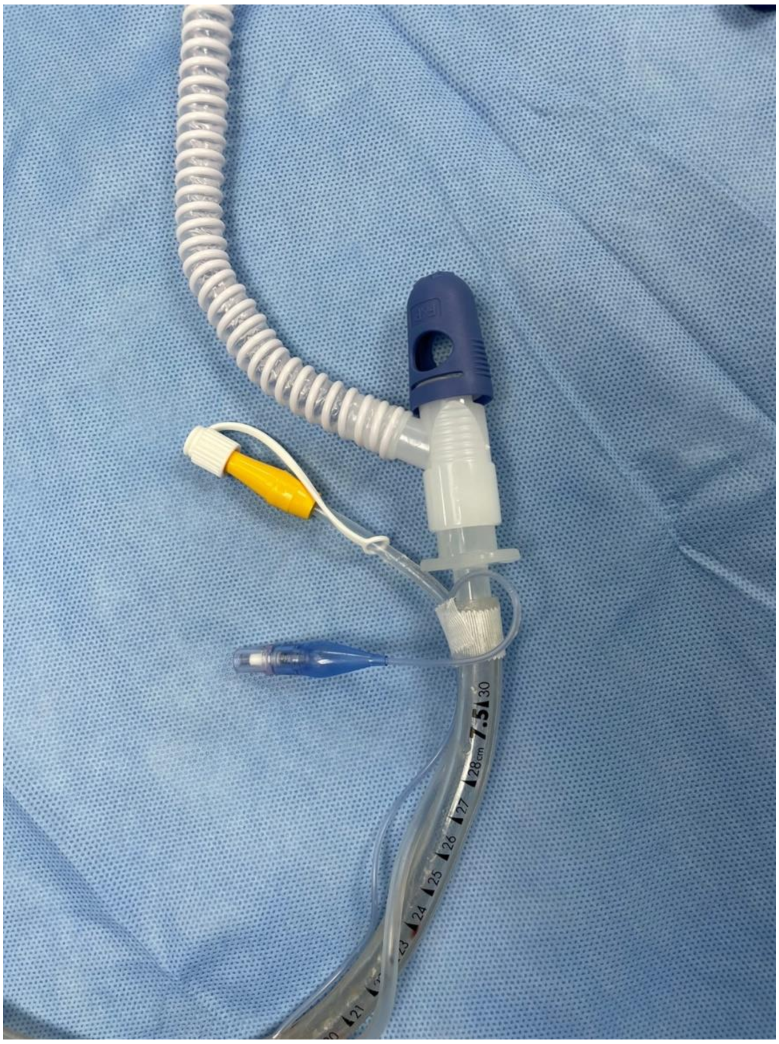

References

1. Boles JM, Bion J, Connors A, Herridge M, Marsh B, Melot C, et al. Weaning from mechanical ventilation. Eur Respir J. 2007;29(5):1033-56.

2. MacIntyre NR, Cook DJ, Ely EW, Jr., Epstein SK, Fink JB, Heffner JE, et al. Evidence-based guidelines for weaning and discontinuing ventilatory support: a collective task force facilitated by the American College of Chest Physicians; the American Association for Respiratory Care; and the American College of Critical Care Medicine. Chest. 2001;120(6 Suppl):375S-95S.

3. Santos Pellegrini JA, Boniatti MM, Boniatti VC, Zigiotto C, Viana MV, Nedel WL, et al. Pressure-support ventilation or T-piece spontaneous breathing trials for patients with chronic obstructive pulmonary disease - A randomized controlled trial. PLoS One. 2018;13(8):e0202404.

4. Chittawatanarat K, Orrapin S, Jitkaroon K, Mueakwan S, Sroison U. An Open Label Randomized Controlled Trial to Compare Low Level Pressure Support and T-piece as Strategies for Discontinuation of Mechanical Ventilation in a General Surgical Intensive Care Unit. Med Arch. 2018;72(1):51-7.

5. Subira C, Hernandez G, Vazquez A, Rodriguez-Garcia R, Gonzalez-Castro A, Garcia C, et al. Effect of Pressure Support vs T-Piece Ventilation Strategies During Spontaneous Breathing Trials on Successful Extubation Among Patients Receiving Mechanical Ventilation: A Randomized Clinical Trial. JAMA. 2019;321(22):2175-82.

6. Teixeira SN, Osaku EF, Costa CR, Toccolini BF, Costa NL, Candia MF, et al. Comparison of Proportional Assist Ventilation Plus, T-Tube Ventilation, and Pressure Support Ventilation as Spontaneous Breathing Trials for Extubation: A Randomized Study. Respir Care. 2015;60(11):1527-35.

7. Thille AW, Coudroy R, Nay MA, Gacouin A, Demoule A, Sonneville R, et al. Pressure-Support Ventilation vs T-Piece During Spontaneous Breathing Trials Before Extubation Among Patients at High Risk of Extubation Failure: A Post-Hoc Analysis of a Clinical Trial. Chest. 2020;158(4):1446-55.

8. Fossat G, Nay MA, Jacquier S, Desmalles E, Boulain T. High-flow oxygen during spontaneous breathing trial for patients at high risk of weaning failure. Intensive Care Med. 2021;47(8):916-7.

9. Burns KEA, Agarwal A, Bosma KJ, Chaudhuri D, Girard TD. Liberation from Mechanical Ventilation: Established and New Insights. Semin Respir Crit Care Med. 2022;43(3):461-70.

10. Vallverdu I, Calaf N, Subirana M, Net A, Benito S, Mancebo J. Clinical characteristics, respiratory functional parameters, and outcome of a two-hour T-piece trial in patients weaning from mechanical ventilation. Am J Respir Crit Care Med. 1998;158(6):1855-62.
